# Rationale, Strengths, and Limitations of Real-World Evidence in Oncology: A Canadian Review and Perspective

**DOI:** 10.1093/oncolo/oyac114

**Published:** 2022-06-28

**Authors:** Laurent Azoulay

**Affiliations:** Centre for Clinical Epidemiology, Lady Davis Institute, Jewish General Hospital, Montréal, Québec, Canada; Department of Epidemiology, Biostatistics, and Occupational Health, McGill University, Montréal, Québec, Canada; Gerald Bronfman Department of Oncology, McGill University, Montréal, Québec, Canada

**Keywords:** real-world evidence, real-world studies, oncology, real-world evidence (RWE), randomized controlled trial (RCT)

## Abstract

Randomized controlled trials (RCTs) continue to be the basis for essential evidence regarding the efficacy of interventions such as cancer therapies. Limitations associated with RCT designs, including selective study populations, strict treatment regimens, and being time-limited, mean they do not provide complete information about an intervention’s safety or the applicability of the trial’s results to a wider range of patients seen in real-world clinical practice. For example, recent data from Alberta showed that almost 40% of patients in the province’s cancer registry would be trial-ineligible per common exclusion criteria. Real-world evidence (RWE) offers an opportunity to complement the RCT evidence base with this kind of information about safety and about use in wider patient populations. It is also increasingly recognized for being able to provide information about an intervention’s effectiveness and is considered by regulators as an important component of the evidence base in drug approvals. Here, we examine the limitations of RCTs in oncology research, review the different types of RWE available in this area, and discuss the strengths and limitations of RWE for complementing RCT oncology data.

## Teaser

There is a growing awareness of the importance of real-world evidence (RWE) in understanding its application to management and informing treatment choices in oncology. This article provides an overview of the strengths and limitations of randomized controlled trial and how it complements RCT oncology data, providing oncologists with additional information to make optimal clinical decisions for their patients.

**Implications for Practice**

There is a growing awareness of the importance of RWE in understanding its application to management and informing treatment choices in oncology. This article provides an overview of the strengths and limitations of RWE and how it complements RCT oncology data, providing oncologists with additional information to make optimal clinical decisions for their patients.

## Introduction

Randomized controlled trials (RCTs) remain the gold standard for evaluating the efficacy of cancer therapies. Their design, however, limits the amount and type of information they can. For clinicians, researchers, and regulators to develop a more complete and in-depth understanding of therapeutic agents and their safety profiles, data obtained from real-world evidence (RWE) are essential to complement the data obtained from RCTs. RWE encompasses data obtained through various research types, essentially anything that is not a traditional RCT.^1^ The US Food and Drug Administration (FDA) defines RWE as “the clinical evidence regarding the usage, and potential benefits or risks, of a medical product derived from analysis of real-world data.”^2^ Potential sources include electronic health records, claims and billing data, disease- or product-specific registries, and digital health solutions outside of conventional clinical trials.^2^ Regulators worldwide have acknowledged RWE as a key component of the evidence base to review in the approval of novel interventions, both during initial approval and for line extensions.^2-5^

The goals of this review are to discuss the rationale for the inclusion of RWE as an essential component of the oncology evidence base; to examine the official positions of various regulatory bodies around the world, including Health Canada and associated decision-makers in Canada; and to provide a high-level overview of the strengths and limitations of RWE. The latter goal is addressed through an examination of questions that can be answered with RWE that cannot be adequately addressed by RCTs, with examples from the literature; and a review of biases that are inherent to RWE, with a brief explanation of methodological approaches that are used to overcome these biases.

## Materials and Methods

A literature search was conducted using PubMed with various combinations of the following terms: “real-world evidence,” “RWE,” “real-world data,” “RWD,” “real-world outcomes,” “real-world populations,” “registry,” “observational studies,” “cancer,” “oncology” and “tumor”. The range of publication dates used in the search was from January 2016 to March 2021. A review of these publications and their references led to the inclusion of studies and commentaries that fell before the initial date range. Relevant national and international regulatory websites, including Health Canada and the US FDA, were also reviewed for regulations related to RWE. The author reviewed the collected publications, developed the framework of the current paper, and selected the publications used to illustrate the key points of the review.

### Rationale for RWE: Essential Complementary Source of Evidence

RCTs form the evidence-based foundation describing the efficacy of interventions, defined as “the extent to which, under ideal circumstances, an intervention produces more benefit than harm.”^6^ Due to their selective populations and highly protocolized nature, applying the findings of RCTs to real-world populations can be challenging. The key shortcomings of RCTs include narrow inclusion criteria, which limit the generalizability to entire patient populations with the malignancy being studied; highly specific treatment regimens, which limit the generalizability to the complex and highly variable treatment regimens used in clinical practice; recruitment difficulties, particularly in rare cancers or less common patient subgroups (eg, those with rare driver mutations); challenges with feasibility or ethics; and an inability to adequately assess an intervention’s safety.^7-9^

RWE is based on longer observation periods in larger populations and can provide complementary evidence to comprehensively describe benefits and risks and improve evidence-based patient care.^1,10-12^ Furthermore, innovations in study designs and analytic methods render RWE better equipped to assess effectiveness. Examples of recent real-world studies that have been impactful in changing clinical practice or guiding regulatory decision-making in oncology are listed in Table 1.

**Table 1.** Selected examples of recent real-world studies with implications in clinical practice or regulatory decision making in oncology

Study	Study population/ intervention	Setting	Research question^a^	Evidence	Implications in clinical practice or regulatory decision making
Khosrow-Khavar et al (2020)^13^	Women with breast cancer treated with aromatase inhibitors or tamoxifen (*n* = 17 922)	Population-based cohort study using the UK Clinical Practice Research Datalink linked to the Hospital Episode Statistics and Office for National Statistics databases	Accurate evaluation of cardiovascular risks of aromatase inhibitors	Aromatase inhibitors were associated with increased risks of heart failure (HR, 1.86 [95% CI, 1.14-3.03]) and cardiovascular mortality (HR, 1.50 [95% CI, 1.11-2.04]) compared with tamoxifen	Real-word evidence of cardiovascular risks and benefit-to-risk ratio of using aromatase inhibitors to treat breast cancer
Liu et al (2021)^14^	Patients with aNSCLC (*n* = 61 094)	Real-world data using the computational framework of Trial Pathfinder	Effect of common inclusion/exclusion criteria on cancer trial populations and outcomes	Common inclusion/exclusion criteria in aNSCLC do not significantly alter HRs for OS: (a) Laboratory tests: blood pressure, albumin levels, lymphocyte count, and neutrophil count (b) Previous treatments: ALK inhibitors, PD-L1 inhibitors, EGFR inhibitor, CYP34A therapies, systemic, or antineoplastic therapies	Design of more inclusive trials and expansion of patient populations that could benefit from new interventions
Luke et al (2019)^15^	Patients with BRAF-mutated metastatic melanoma (*n*=60)	Retrospective, observational real-world study using patient-level data from Cardinal Health Oncology Provider Extended Network	Role of treatment sequence on patient outcomes	The sequence of targeted therapy followed by immunotherapy was associated with higher response rate and longer treatment duration compared to the opposite sequence as first-line therapy	Real-world evidence of a preferred sequence of targeted therapy followed by immunotherapy as first-line therapy for BRAF-mutated metastatic melanoma
Kraus et al (2022)^16^	Men with metastatic breast cancer (*n* = 20)	Retrospective real-world analysis of pharmacy and medical claims data from a US database (IQVIA Inc.) and health records from the Flatiron Health database	Efficacy of first-line palbociclib in combination with ET in a rare population	Combination of palbociclib + ET was associated with a longer median duration of therapy (9.4 vs. 3.0 months) and higher response rate (33.3% vs 12.5%)compared to ET alone	Real-word evidence supporting the approval of palbociclib for use in combination with ET to treat previously untreated male patients with metastatic breast cancer
Gökbuget et al (2016)^17^	Adult patients with BCP ALL	Retrospective analysis of historical datasets from Europe and the US	Efficacy of blinatumomab in a rare population	Blinatumomab treatment was associated with increased odds of complete response (OR, 2.68; 95% CI, 1.67-4.31) and improved OS (HR, 0.536; 95% CI, 0.394-0.730) compared to historical standard therapy	Real-word evidence supporting the approval of blinatumomab for the treatment of patients with BCP ALL in first or second complete remission

Research question that could not be reliably addressed by randomized clinical trials.

Abbreviations: AEs, adverse events; ALP, alkaline phosphatase; ALT, alanine aminotransferase; aNSCLC, advanced non–small cell lung cancer; AST, aspartate aminotransferase; BCP ALL, B-cell precursor acute lymphoblastic leukemia; CI, Confidence interval; ET, endocrine therapy; HR, hazard ratio; OR, odds ratio; OS, overall survival.

### Limitation of RCTs to Define the Safety of an Intervention

RCTs are time-limited and often not designed or powered to assess safety outcomes. Furthermore, the strict eligibility criteria for RCTs are often designed in part to limit the likelihood of the participants experiencing adverse events (AEs).^18^ As a consequence, the rates of AEs reported in RCTs may be much lower than among patients treated in clinical practice.

The use of RWE is critical for the comprehensive evaluation of the safety of any given intervention. RWE can evaluate medications over longer periods of time in larger and more diverse groups of subjects than can be enrolled in clinical trials.^9^ This not only allows for better clarity on the expected frequency of common AEs but also allows for the identification and quantification of rare AEs.^9^ An example of an important safety consideration with cancer therapy is the reported potential for increased cardiovascular risk among women with breast cancer treated with aromatase inhibitors. Clinical trials, meta-analyses, and initial real-world studies investigating this safety outcome have generated conflicting results.^19-25^ A population-based cohort study using UK data linked the Clinical Practice Research Datalink to the Hospital Episode Statistics and Office for National Statistics databases to identify 23 525 patients newly diagnosed with breast cancer treated with either an aromatase inhibitor or tamoxifen.^13^ In this study, aromatase inhibitors were associated with increased risks of heart failure and cardiovascular mortality compared with tamoxifen, with nonsignificant increased risks of myocardial infarction and ischemic stroke. To further investigate the impact of sequencing, the same group investigated the cardiotoxicity of aromatase inhibitors as second-line treatment after tamoxifen compared to continued tamoxifen treatment, using the same real-world databases.^26^ In this analysis, aromatase inhibitors were associated with a significant 2-fold higher risk of myocardial infarction compared to continued tamoxifen. The hazard ratios were elevated for ischemic stroke and heart failure but were not statistically significant. These large, well-designed, real-world studies have provided additional insight on this critical element of the benefit-to-risk assessment with these commonly used agents.

### Limited Generalizability (External Validity) of RCTs

#### Study Population

The design requirements of RCTs include specific constraints on the study population and may exclude patients who might also benefit from the intervention. For example, an analysis of individuals with 11 common malignancies who were enrolled in the Alberta Cancer Registry between 2004 and 2015 showed that, among the more than 125 000 patients in the registry, 38% were considered trial-ineligible.^27^ This assessment of eligibility was based on exclusion criteria that are common to most oncology trials: advanced age (>75 years), the presence of anemia, the presence of comorbid heart disease, uncontrolled diabetes, kidney disease, or liver disease, or history of a prior malignancy or immunosuppression (Table 2).^27^ With common malignancies, the proportion of the overall patient population who participate in clinical trials is very low; in breast cancer, for example, it has been estimated that only 3% of patients participate in clinical trials.^28^

**Table 2.** Reasons for trial non-eligibility among cancer patients in the Alberta Cancer Registry, 2004-2015 (*N* = 125 316)^27^

Reason	*n* (%)
Age >75 years	30 661 (25%)
Presence of heart disease	10 996 (16%)
Kidney disease	6840 (5%)
Uncontrolled diabetes	5984 (5%)
Liver disease	4778 (4%)
Abnormal bloodwork	2339 (2%)
Prior malignancy	1872 (1%)
Any immunosuppression	1642 (1%)

Real-world studies can be designed to complement the findings of RCTs in a more inclusive population, encompassing individuals who would not have been eligible for RCTs.^7-9^ For example, a real-world study using a large US healthcare database (Flatiron Health) compared the use of eligibility criteria from 10 major clinical trials to a much more inclusive set of criteria on overall survival (OS) rates in 61 094 patients with advanced non-small cell lung cancer (aNSCLC).^14^ By relaxing the eligibility criteria to only those with a definitive impact on OS, the investigators reported that the pool of patients more than doubled on average across the studies (from an average of 1553 patients eligible to an average of 3209). These findings suggest that patients not eligible under the original trial criteria could benefit from the interventions.^14^ Furthermore, the analysis provided vital information for clinical practice, as the researchers identified the particular eligibility criteria that did not have a substantial impact on the hazard ratio for OS (Table 1).^14^ However, this does not preclude the possibility that the factors used in inclusion or exclusion criteria, such as response to prior treatment, could affect treatment outcomes. Collectively, these examples highlight that RWE may allow clinicians and regulators to reliably and accurately extrapolate the findings of pivotal clinical trials to broader populations of patients who may benefit from the treatment in question.

### Treatment Regimens

Given that most RCTs are designed to evaluate a single intervention against a particular control, their designs limit the heterogeneity of treatment regimens within the trial setting.^6^ This does not reflect the complexity of care in the real world, including the reality of differing and evolving treatment sequences and modalities.^29^ RCTs often compare a new drug with the standard of care. However, in oncology, the standard of care for a given malignancy or subpopulation can change rapidly.^30,31^ Moreover, standard of care interventions can differ across countries, regions, or centers, often based on reimbursement and access issues.^32^

Properly conducted real-world research can address these potential discrepancies to complement the RCT evidence base. This can include assessments of the comparative effectiveness of different drugs or regimens, providing a more realistic look at interventions in the context of a given country, region, or center. For example, an analysis of the large real-world US SEER-Medicare database investigated treatment sequences in 6639 patients with metastatic breast cancer (MBC).^33^ The heterogeneity of sequencing in real life was illustrated by their finding that 56% of these patients received a sequence that fewer than 11 other patients received.^33^ This makes it challenging to extrapolate the results of RCTs with defined sequencing to the real world. RWE can help clinicians and regulators effectively conceptualize treatment patterns and patient outcomes, improving patient care in oncology.

The treatment landscape for BRAF-mutated metastatic melanoma is rapidly evolving. In the US, 7 new therapies were approved for this indication from 2014 to 2018, making it difficult to evaluate optimal sequences using data from RCTs. A retrospective, real-world observational study was designed using patient-level data from Cardinal Health Oncology Provider Extended Network (OPEN), a community of over 7000 oncologists from across the US.^15^ This analysis of 600 patients suggested that the sequence of targeted therapy followed by immunotherapy was associated with a higher response rate and longer treatment duration compared to the opposite sequence as first-line therapy for BRAF-mutated metastatic melanoma.^15^ While these observations have their limitations, this type of RWE can help bridge a knowledge gap left by the RCTs.

### Investigation of Areas Where RCTs Are not Feasible or Are Unethical

There are also settings where large RCTs may not be possible; chief among these are rare diseases or subgroups of patients with uncommon molecular profiles.^9^ Finding sufficient numbers of patients to power a traditional phase III RCT for these populations may not be feasible over an acceptable amount of time. As such, RWE may be the only way to reliably evaluate therapies in these scenarios.^9,34^ An increasingly common way to approach these scenarios is to conduct a single-arm open-label study of the investigational intervention, using a control arm from real-world sources.

For example, male breast cancer is a very small subset of the overall breast cancer population (approximately 1%).^35^ Conducting large RCTs for interventions in this population has not been successful, and extrapolating data from the much larger populations of women with this malignancy may not be appropriate.^36^ Palbociclib is a cyclin-dependent kinase (CDK) 4/6 inhibitor that was approved for use in hormone receptor (HR)-positive, human epidermal growth factor receptor 2 (HER2)-negative advanced or metastatic breast cancer based on a clinical trial that excluded men.^37-42^ To investigate the efficacy of this therapy in men, investigators used 2 parallel approaches. Treatment patterns in men, including duration of therapy, were described through a retrospective analysis of pharmacy and medical claims data from a US database (IQVIA Inc), while real-world clinical response was evaluated by analyzing health records from the Flatiron Health database.^16^ These analyses showed that the combination of palbociclib plus endocrine therapy (ET) was associated with a longer median duration of therapy compared to ET alone (9.4 vs. 3.0 months) and that the response rate was also higher (33.3% vs. 12.5%; Table 1).^16^ Although the cohort size in this study was small (12 patients in the palbociclib plus ET group and 8 patients in the ET alone group), these data provided clinically important information for this uncommon breast cancer subgroup that had not been available from RCTs and contributed to the line extension of palbociclib for use in combination with endocrine therapy to treat previously untreated male patients with MBC.

RWE also has a key role to play where clinical trials may be unethical.^43^ For example, one could not design an RCT with a placebo arm in clinical scenarios where depriving subjects of a reasonable standard of care would cause significant harm. In RWE, however, one could use database resources to elucidate differences between groups who did or did not receive a particular treatment regimen.

### Complementary Support for Drug Development and Regulatory Approval

In Canada and around the world, there is a growing recognition of the need for and utility of RWE to support, extend, complement, or, in some cases, substitute for clinical trial efficacy and safety outcomes during drug development and both pre- and post-marketing regulatory approval.^2-5,23,44-47^ RWE has been successfully used in submissions of cancer therapies to various regulatory bodies worldwide.^45,46^ For example, in 2017, Health Canada approved avelumab for the treatment of Merkel cell carcinoma, with the decision based, in part, on the inclusion of historical controls from RWE, which complemented phase II, single-arm, open-label data.^46^ Similarly, in the case of palbociclib for male breast cancer, the above-mentioned real-life data were successfully used in an FDA submission for a line extension for palbociclib for this indication in the US.^48^

Efforts have been made across jurisdictions to codify and regulate the use of RWE for such purposes. In the US, the FDA has published guidelines detailing the circumstances under which manufacturers can use RWE to support the approval of a medicine.^2^ Similarly, the European Medicines Agency (EMA) has detailed its views on this topic.^3^ In Canada, Health Canada has partnered with other key organizations, including the pan-Canadian Oncology Drug Review (pCODR) at the Canadian Agency for Drugs and Technologies in Health (CADTH) and the Institut national d’excellence en santé et en services sociaux (INESSS) in Québec, to optimize the use of RWE for regulatory decisions.^4^ The official Health Canada notification lists 3 scenarios for which RWE submissions are encouraged: (1) to expand evidence-based indications for populations frequently excluded from clinical studies; (2) for drugs or diseases where clinical studies are unfeasible (eg, rare diseases); and (3) for drugs or diseases where clinical trials are considered unethical (eg, to extrapolate dosages from animal studies to treat humans exposed to chemical or biological threats during emergencies).^5^

In parallel, in recognition of the potential values of RWE, a more inclusive group of Canadian stakeholders was formed to address challenges and establish a framework for Canadian provinces regarding the generation and use of RWE for cancer drug funding decision-making.^44^ The Canadian Real-world Evidence for Value of Cancer Drugs (CanREValue) collaboration includes representatives from Health Canada, provincial ministries and departments of health, health technology assessment organizations, provincial cancer agencies, the Canadian Association of Provincial Cancer Agencies, the pan-Canadian Pharmaceutical Alliance, and the Patented Medicine Prices Review Board. The group also includes applied researchers, clinicians, and patient and family representatives. There are 5 separate working groups within CanREValue, each tasked with focusing on specific processes in the generation and use of RWE: (1) planning and drug selection; (2) methods; (3) data; (4) reassessment and uptake; and (5) engagement.^44^

### Biases in Real-world Studies and Methods to Mitigate Them

The codification of criteria for acceptable RWE by regulatory bodies and the establishment of working groups like CanREValue reflect the reality that there is substantial heterogeneity in the quality of real-world study studies and, therefore, the applicability of their findings to clinical decision-making and regulatory review. RCTs are not without their own biases.^49^ Because of the observational nature of real-world studies, they are inherently more prone to certain biases, such as confounding.^44^ While it is essential for clinicians, researchers, and regulators to understand the potential biases in generating RWE and the impact these biases can have on findings if they are not adequately addressed in study design, providing a complete description and dissection of each of these is beyond the scope of this review. However, the following section provides a high-level overview of biases found in real-world studies, including biases associated with the selection of comparator and those related to exposure, timing, and outcomes. For each of these, methodological approaches that can be used to mitigate these biases are briefly discussed.

### Common Biases in Real-world Studies

There are several potential biases that need to be taken into account when designing or interpreting real-world studies. These include biases involved in the selection of comparators, issues of temporality, measurement of drug exposure, and methods of adjustment when comparing real-world evidence to clinical trial data.^50^

With respect to comparators, selecting an active comparator may make it easier to design a real-world comparative study to emulate an RCT. If there is no active comparator incorporated into the study, the goal is typically to create a control arm that reflects the current standard of care; this leads to inherent differences in patient characteristics between groups, which must be accounted for in the trial design.^50^ Regardless of whether there is an active comparator, selection bias and confounding by indication or severity are key challenges to consider in trial design.^51^

Temporality is another potential source of bias for real-world studies that needs to be addressed in study design. Many malignancies have rapidly evolving treatment landscapes; thus, the time period of study for both the intervention and the controls needs to be constructed to take this into account. Furthermore, the use of a historical control may not adequately represent current standards of care; historical controls may still be suitable for rare diseases, where there are few new treatments available.^50^

The particular time-related biases are well described in the literature and include reverse causality, immortal time bias, time-lag bias, time-window bias, and immeasurable time bias.^52^ A recent review of observational studies of new indications for older drugs found that there were many findings of unrealistic effectiveness that were influenced by avoidable time-related biases.^52^ These inappropriate findings could have been avoided with proper design and analysis.^52^

Real-world studies must also consider issues in design related to drug exposure. For example, it is important to determine whether the drug has an acute or chronic (cumulative) effect on the outcome. This assessment should be informed by what is known about the natural history of the disease. For acute outcomes (eg, myocardial infarction), the latency period is typically assumed to be short (ie, days to months); in contrast, the latency period for slow-developing outcomes (eg, cancers) is usually long (ie, months to years).

With respect to the definition of the exposed population, the gold-standard approach in RCTs is intention-to-treat, which assumes patients remain continuously exposed to the study drugs until the end of follow-up and helps preserve randomization. However, in real-world research, alternate definitions of exposure should be considered. For example, in the population-based analysis comparing the cardiovascular safety of aromatase inhibitor and tamoxifen, the investigators used an “on-treatment” definition of exposure in which the patients were followed while continuously exposed to the study drugs.^13^ In this definition, patients were censored at discontinuation of the initial treatment or at a switch between tamoxifen and aromatase inhibitors (or vice versa). A grace period was also included beyond the discontinuation to account for residual drug effects and incomplete adherence to treatment.

Other methods of defining exposure include the time-varying approach, as well as hybrid methods incorporating elements of more than one approach. Certain approaches are best suited for certain outcomes, and each approach has its strengths and limitations. To demonstrate the suitability of the selected approach (eg, to assess assumptions related to the length of grace and washout periods), sensitivity analyses should be conducted. There are also several outcome-related biases to be addressed in the design of real-world studies, including detection bias, insidious outcomes, overly broad outcome definitions, and assessment of validity.

### Methodological Approaches to Mitigate Biases

There are many methodological approaches to mitigating biases in RWE, and which method is most appropriate depends on the research question. Propensity scores (PS) reflect the patients’ predicted probability of receiving a certain treatment given their characteristics and have emerged as a cornerstone of confounding adjustment in observational studies.^53,54^ Using PS-based methods, researchers can target causal inference in observational studies, similar to randomized studies, by measuring the differences in outcomes between treated and reference populations.

PS methods include matching, stratification, adjustment as a regressor, and weighting.^53,54^ While PS matching has been a popular approach in the past, this method has the important limitation of discarding unmatched observations (particularly those in the control group) and requires a very large pool of patients in the control group, making this method suboptimal when investigating an uncommon exposure or a rare outcome. Other PS methods can avoid these limitations. Unlike matching, weighting offers more precision by keeping most observations in the analysis and can facilitate clearer reporting of the balance between the treatment and reference groups. Weighting is also far more flexible, with multiple available variations allowing the targeting of specific populations. Specific traditional approaches to PS weighting include inverse probability treatment weights (IPTW) and standardized mortality ratio weights (SMRW). Newer approaches such as fine stratification weights, matching weights, and overlap weights may overcome some of the limitations of traditional weighting approaches.^53^ However, these different methods of controlling for confounding bias are not interactable and generate different estimands, which may impact the interpretation of the research findings. For example, the estimand for PS matching is the average treatment effect in the treated (ATT), while IPTW and fine stratification weights provide measures of the average treatment effect in the population (ATE).^55,56^

For pragmatic studies that attempt to emulate the comparative nature of an RCT using real-world methods, an increasingly popular method is the “new user, active comparator design.”^57^ This design offers the theoretical advantage of mitigating confounding bias by indication, healthy users, and frailty at the design stage. The key principles of this method are that the active comparator component restricts inclusion in the study to those subjects who have an indication for treatment without contraindications (including frailty). The new user (NU) component mitigates bias by aligning individuals at a uniform point in time to start follow-up (ie, treatment initiation), which helps to ensure appropriate temporality between covariate and exposure assessment.^57^

While the new user, active comparator design has been embraced for studies comparing one intervention to another (eg, comparison of aromatase inhibitors to tamoxifen in breast cancer), other causal questions are best addressed with other research methodologies. For example, for studies investigating whether to switch treatment or keep the patient on the existing treatment, such questions are best answered with novel designs, such as the “prevalent new-user design.”^58^ This approach addresses key comparator and temporality-related biases, allowing for more reliable comparisons between newer and older drugs, including those who switch from the old to the new drug.^58^ This approach was used in the analysis cited above comparing the continuation of tamoxifen therapy with a switch to an aromatase inhibitor.^26^

## Conclusions

Data obtained from real-world studies have an integral role in evidence-based medicine, serving as an essential source of safety information and a complement to efficacy data from RCTs. RWE is particularly useful for expanding the evidence base to encompass populations of patients who are not well represented in RCTs but who may benefit from the interventions in question. RWE is also critical in the setting of complex or rapidly evolving treatments, where RCT design cannot answer all the relevant questions. Defining the role of treatments for rare cancers or rare subtypes is another key function of RWE. RWE is essential to better defining a treatment’s safety, particularly over the long term. These uses enhance clinical knowledge and patient care with approved agents. Moreover, RWE is becoming widely accepted by regulators for new drug approvals or line extensions. While RWE has many uses, it also has many limitations. Efforts are being made by regulators and other groups to develop best practices for the mitigation of common biases in the design of real-world studies.

## Data Availability

Data analyzed in this study were a re-analysis of existing data, which are openly available at locations cited in the reference section.
